# Formate-driven H_2_ production by whole cells of *Thermoanaerobacter kivui*

**DOI:** 10.1186/s13068-022-02147-5

**Published:** 2022-05-11

**Authors:** Yvonne Burger, Fabian M. Schwarz, Volker Müller

**Affiliations:** Department of Molecular Microbiology and Bioenergetics, Institute of Molecular Biosciences, Johann Wolfgang Goethe University, Max-von-Laue-Str. 9, 60438 Frankfurt, Germany

**Keywords:** Biohydrogen, Dark fermentation, Acetogenic bacteria, Bioreactor, qH2, HER, Optimization, Scale-up, Hydrogen-dependent CO_2_ reductase (HDCR)

## Abstract

**Background:**

In times of global warming there is an urgent need to replace fossil fuel-based energy vectors by less carbon dioxide (CO_2_)-emitting alternatives. One attractive option is the use of molecular hydrogen (H_2_) since its combustion emits water (H_2_O) and not CO_2_. Therefore, H_2_ is regarded as a non-polluting fuel. The ways to produce H_2_ can be diverse, but steam reformation of conventional fossil fuel sources is still the main producer of H_2_ gas up to date. Biohydrogen production via microbes could be an alternative, environmentally friendly and renewable way of future H_2_ production, especially when the flexible and inexpensive C1 compound formate is used as substrate.

**Results:**

In this study, the versatile compound formate was used as substrate to drive H_2_ production by whole cells of the thermophilic acetogenic bacterium *Thermoanaerobacter kivui* which harbors a highly active hydrogen-dependent CO_2_ reductase (HDCR) to oxidize formate to H_2_ and CO_2_ and vice versa. Under optimized reaction conditions, *T. kivui* cells demonstrated the highest H_2_ production rates (qH_2_ = 685 mmol g^−1^ h^−1^) which were so far reported in the literature for wild-type organisms. Additionally, high yields (*Y*_(H2/formate)_) of 0.86 mol mol^−1^ and a hydrogen evolution rate (HER) of 999 mmol L^−1^ h^−1^ were observed. Finally, stirred-tank bioreactor experiments demonstrated the upscaling feasibility of the applied whole cell system and indicated the importance of pH control for the reaction of formate-driven H_2_ production.

**Conclusions:**

The thermophilic acetogenic bacterium *T. kivui* is an efficient biocatalyst for the oxidation of formate to H_2_ (and CO_2_). The existing genetic tool box of acetogenic bacteria bears further potential to optimize biohydrogen production in future and to contribute to a future sustainable formate/H_2_ bio-economy.

**Supplementary Information:**

The online version contains supplementary material available at 10.1186/s13068-022-02147-5.

## Background

Molecular hydrogen is considered as an attractive, alternative energy carrier which can be produced environmentally friendly and renewable. If hydrogen is produced from renewable energy sources by water splitting, no net CO_2_ is generated in the production process and H_2_ can be regarded as a promising environmentally friendly fuel [1, 2]. However, traditional routes such as steam reforming and partial oxidation of coal, oil and natural gas are still the main producers of H_2_ gas to date [3, 4]. Therefore, the production of H_2_ from renewable, sustainable and environmentally friendly sources become more and more important. Biohydrogen production via microbial organisms has already been studied extensively over several decades, but is still an active field of research which comprises three major categories: (1) biophotolysis (direct and indirect), (2) photofermentation, and (3) dark fermentation [5–7]. In the latter one, the organisms do not rely on the availability of light and can utilize a vast variety of carbon sources to break them down into H_2_ and most likely organic acids and alcohols. Since dark fermentative H_2_ production has high production rates and does not need direct solar input, the process possesses a greater potential for practical applications [8, 9]. Here, formic acid/formate is a very flexible and inexpensive substrate for H_2_ production by fermentative microbes [7, 10]. The hydrogen content in formic acid is 4.4 wt% and the compound is regarded as liquid organic hydrogen carrier (LOHC) as well as feedstock chemical and microbial carbon source [11, 12]. Natural formatotrophic microorganisms are able to assimilate formate in their metabolism using either the Calvin cycle, the serine pathway or the Wood–Ljungdahl pathway (WLP) [12, 13]. Several acetogenic bacteria (i.e., *Acetobacterium* *woodii*, *Clostridium* *ljungdahlii* and *Moorella* *thermoacetica*) and methanogens can utilize formate via the WLP as sole energy and carbon source for growth [14, 15]. However, different microorganisms, especially of the *Thermoanaerobacterales*, *Clostridiaceae* and *Enterobacteriaceae* families are known to oxidize formate with the concomitant evolution of H_2_ and CO_2_ [8] and diverse enzyme systems are involved to catalyze the reaction. The enterobacterium *Escherichia coli* has become a workhorse for enhanced biohydrogen production due to a formate hydrogen lyase (FHL) system which catalyzes the disproportionation of formate to H_2_ and CO_2_ (Eq. 1) [16–18]:

1 $$HCOO^{ - } + H_{2} O \leftrightarrow HCO_{3}^{ - } + H_{2} \quad \Delta G^{{0^{\prime}}} = + 1.3 {\text{kJ mol}}^{ - 1}$$

The purified FHL consists of a membrane-associated [NiFe]-hydrogenase, a molybdenum-containing formate dehydrogenase as well as several electron-transferring iron–sulfur proteins [19]. The good genetic accessibility of the organism as well as different strategies such as heterologous gene expression, metabolic engineering, adaptive evolution and protein engineering were already applied to enhance H_2_ production by *E. coli* [20–24]. Another example for hydrogen production from formate is the hyperthermophilic archaeon *Thermococcus* *onnurineus*. This archaeon has a similar membrane-bound enzyme complex as *E. coli* to mediate formate-driven H_2_ production which is called Fdh-Mrp-Mbh enzyme complex. The enzyme consists of a formate dehydrogenase (Fdh) module, a membrane-bound hydrogenase (Mbh) module and a multisubunit Na^+^/H^+^ antiporter (Mrp) module. So far, the entire enzyme complex could not be purified from *T. onnurineus*, but the enzyme is involved in chemiosmotic energy conservation [25–27].

Recently, a soluble, biotechnological interesting enzyme complex that is involved in formate metabolism of acetogenic bacteria was purified from the thermophilic acetogenic bacterium *Thermoanaerobacter kivui*, named as hydrogen-dependent CO_2_ reductase [28]. The enzyme complex consists of a formate dehydrogenase and a [FeFe]-hydrogenase subunit which are, most likely, connected by two electron-transferring, iron–sulfur subunits. So far, the HDCRs were only purified from the thermophilic *T. kivui* and the mesophilic acetogenic bacterium *A. woodii*, but bioinformatic analysis of available genome data indicates the presence of HDCR-like enzymes also outside of this bacterial group [28, 29]. The purified and characterized HDCR enzymes catalyze the direct hydrogenation of CO_2_ to formic acid with remarkable catalytic rates, outcompeting chemical catalysts under comparable moderate reaction conditions [30]. But the enzymes do not only catalyze CO_2_ reduction (H_2_:CO_2_ oxidoreductase activity), moreover, they catalyze the reverse reaction of formate oxidation (formate: H^+^ oxidoreductase activity) with almost identical rates. The thermophilic enzyme from *T. kivui* showed a H_2_:CO_2_ oxidoreductase activity and a formate: H^+^ oxidoreductase activity of 900 and 930 U/mg [28], respectively, demonstrating the reversible nature of the enzyme and the high potential of the organism in formate-based H_2_ production. It is worth mentioning that the catalytic rates of the thermophilic HDCR are almost two orders of magnitude higher than the mesophilic enzyme from *A. woodii* [28].

For biotechnological applications, the HDCR-based conversion efficiency of H_2_ and CO_2_ to formate was already proven by resting cells of *T. kivui* and *A. woodii* in serum bottle and bioreactor scale [29, 31, 32]. But formate-driven H_2_ production was so far only investigated for *A. woodii* cells [32, 33]. To take advantage of the multiple times higher reaction speed of the thermophilic enzyme, whole-cell catalysis for biohydrogen production from formate using the thermophilic acetogen *T. kivui* was addressed in this study.

## Results

### H_2_ production with resting cells

**Fig. 1:**
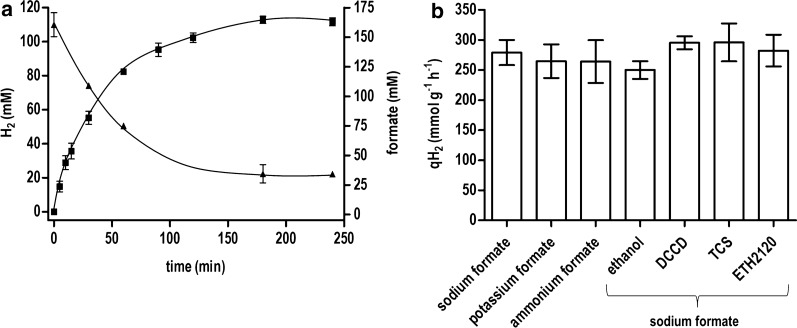
H_2_ production from formate by resting *T. kivui* cells in presence or absence of metabolic inhibitors. Resting cells (0.6 mg mL^−1^) were added to preheated (60 °C) imidazole buffer (50 mM imidazole, 20 mM MgSO_4_, 20 mM KCl, 2 mM DTE, 4 µM resazurin, pH 7) under a N_2_ atmosphere. **a** The reaction was started by addition of 150 mM of sodium formate. H_2_ (black squares) and formate concentrations (black triangles up) are plotted against time. **b** 30 µM DCCD, TCS or ETH2120 or 20 µL ethanol was added to the serum bottles 10 min before the reaction was started by adding 150 mM of sodium, potassium or ammonium formate as indicated. The specific hydrogen production rate was calculated based on the first 15 min after start of the reaction. All data points are mean ± SD, *N* = 2

To investigate the formate-driven H_2_ production potential of resting cells from *T. kivui*, the organism was grown in complex medium with glucose as substrate, cells were harvested and washed, and were subsequently incubated in reaction buffer at a protein concentration of 0.6 mg mL^−1^ (corresponding to 1.54 mg_CDW_ mL^−1^). After the addition of 150 mM sodium formate, H_2_ production as well as substrate and metabolite concentrations were monitored over time (Fig. 1a). The cells started to produce H_2_ with an initial specific H_2_ productivity (qH_2_) of 249 ± 51 mmol g^−1^ h^−1^ (162 mmol g_CDW_^−1^ h^−1^). 1.2 mmol H_2_ was produced from 1.4 mmol formate consumed leading to a yield of H_2_ produced per substrate consumed (*Y*_(H2/formate)_) of 0.86 mol mol^−1^. Interestingly, only traces of acetate (2.4 ± 2.3 mM) were finally produced. Unlike in *A. woodii* uncoupling agents did not stimulate H_2_ production (Fig. 1b) and did not affect the produced amount of acetate. These results were surprising since growing cultures of *T. kivui* are expected to disproportionate formate to acetate and CO_2_ according to Eq. 2:

2 $$4\;HCOOH \leftrightarrow CH_{3} COOH + 2CO_{2} + 2H_{2} O$$

But in contrast to the expectations, formate was nearly completely oxidized to H_2_ and CO_2_ by resting cells. Therefore, it was of interest to study formate metabolism in growing cells. As shown before [15], *T. kivui* disproportionated formate according to Eq. 2 under different conditions (Additional file 1: Fig. S1). The utilization of 200 mM of formate resulted in the formation of 40 ± 5 mM acetate in average, yielding a stoichiometry of 5:1. This clearly shows, that *T. kivui* cells grew on formate according to Eq. 2. Cell suspensions of formate-grown cells behaved as cell suspensions from glucose-grown cells: from 300 mM of formate only traces of acetate (0.15 ± 0.02 mM) were formed and 160 ± 17 mM of hydrogen were produced (Additional file 1: Fig. S2).

**Fig. 2:**
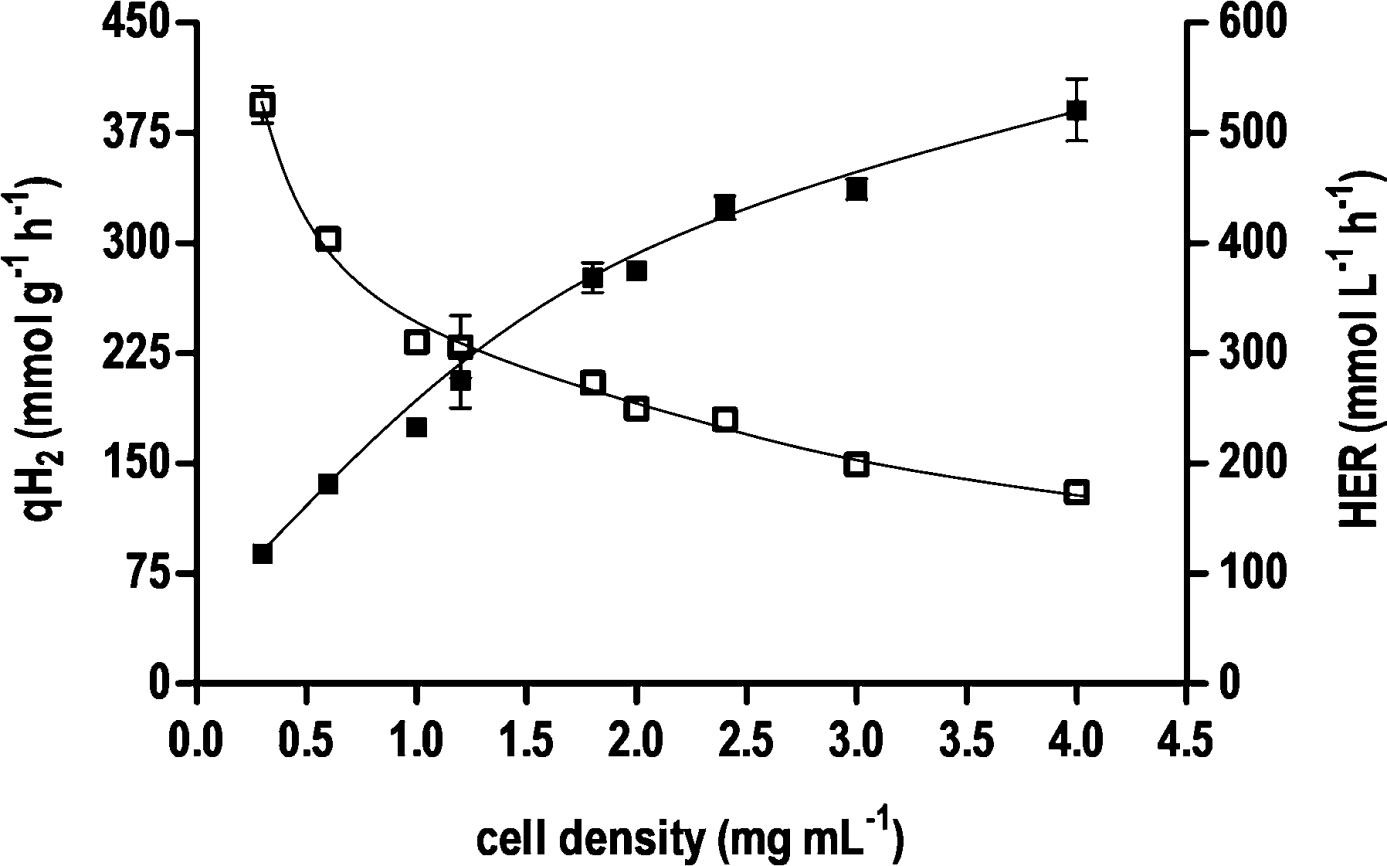
Influence of cell densities on volumetric and specific H_2_ production rates of *T. kivui*. Resting cells (0.3–4 mg mL^−1^) were added to preheated (60 °C) imidazole buffer (50 mM imidazole, 20 mM MgSO_4_, 20 mM KCl, 2 mM DTE, 4 µM resazurin, pH 7) under a N_2_ atmosphere. The reaction was started by addition of 300 mM of sodium formate. Specific H_2_ production rate (open squares) and volumetric H_2_ production rate (black squares) are plotted against the cell densities. The specific H_2_ production rates were calculated based on the first 15 min after start of the reaction. All data points are mean ± SD, *N* = 2

Formate-driven H_2_ production was characterized in detail using glucose-grown *T. kivui* cells. The qH_2_ decreased with increasing pH (Additional file 1: Fig. S3). At a pH range of 5.5–7 the highest qH_2_ of 245 mmol g^−1^ h^−1^ was observed, whereas under alkaline conditions (pH 9) only 29% of the activity was present. Temperature dependence of whole cell catalysis was tested from 30 to 80 °C (Additional file 1: Fig. S4). The temperature profile showed the highest qH_2_ at 70 °C (qH_2_ = 370 ± 84 mmol g^−1^ h^−1^) which is close to the physiological growth temperature optimum of 66 °C of *T. kivui*. But at ambient temperatures of 30 °C, still 11% of the maximum qH_2_ was achieved. In the next experiment, the effect of increasing formate concentrations (25 mM to 8 M) on H_2_ productivity was investigated. Within this range, initial H_2_ production rates were optimal at 600 mM sodium formate and decreased up to 8 M substrate (Additional file 1: Fig. S5). An exponential decrease of the maximum qH_2_ of 394 ± 17 mmol g^−1^ h^−1^ (256 mmol g_CDW_^−1^ h^−1^) at 0.3 mg mL^−1^ was observed with increasing cell densities. On the other hand, the hydrogen evolution rate (HER) increased up to 521 ± 40 mmol L^−1^ h^−1^ at a cell concentration of 4 mg mL^−1^ (Fig. 2).

The storage stability of resting cells, an important parameter in an industrial application, was also tested. Therefore, the qH_2_ of stored (4 °C) *T. kivui* cells were monitored in cell suspension experiments over several weeks. 50% and 25% of the initial qH_2_ was reached after 2.5 and 6 weeks, respectively (Additional file 1: Fig. S6). Another aspect in practical applications is the sensitivity of the catalyst towards oxygen (O_2_), especially when [FeFe]-hydrogenase enzymes are involved in biocatalysis. Here, up to 0.79% O_2_ in the gas atmosphere (corresponding to 5.6 µM O_2_ in liquid phase) still allowed sufficient H_2_ production with a qH_2_ of 210 ± 5 mmol g^−1^ h^−1^. Higher dissolved concentrations of O_2_ led to a dramatic decrease in qH_2_ (Additional file 1: Fig. S7).

**Fig. 3:**
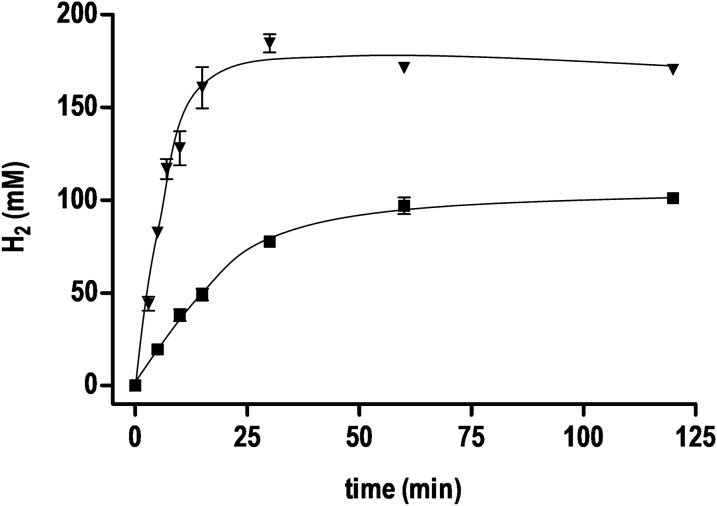
Formate- and cell density-dependent H_2_ production at optimal pH and temperature. Resting cells were added to preheated (70 °C) imidazole buffer (50 mM imidazole, 20 mM MgSO_4_, 20 mM KCl, 2 mM DTE, 4 µM resazurin, pH 7) under N_2_ atmosphere. The reaction was started by adding sodium formate. Black squares, 0.3 mg mL^−1^ and 300 mM sodium formate; black triangles down, 4 mg mL^−1^ and 600 mM sodium formate. All data points are mean ± SD, *N* = 2

Next, we used the optimal pH of 7 and an optimal temperature of 70 °C, but raised the formate and the protein concentration (Fig. 3). At 4 mg mL^−1^ total cell protein and 600 mM formate, H_2_ production was fast but came to completion after 25 min. The plateau reached was only 175 mM H_2_, which is only 58% of the theoretical value. At lower protein and formate concentrations (0.3 mg mL^−1^ total cell protein and 300 mM formate), hydrogen production was slower and came to an end at 100 mM H_2_, again only 67% of the theoretical value. Formate oxidation leads to an alkalinization of the buffer and indeed, at the end of the experiment the pH was around 8.4–8.6. The results of qH_2_ and HER are summarized in Table 1.

**Table 1:** qH_2_ and HER of formate-based H_2_ production by whole *T. kivui* cells under optimized reaction conditions

H_2_ production	^a^Condition: 0.3 mg mL^−1^ cells 300 mM sodium formate	^a^Condition: 4 mg mL^−1^ cells 600 mM sodium formate
qH_2_ (mmol g ^−1^ h ^−1^)	685 ± 87	250 ± 2
HER (mmol L ^−1^ h ^−1^)	205 ± 26	999 ± 6

^a^Cell suspension experiment in imidazole buffer (50 mM imidazole, 20 mM MgSO_2_, 20 mM KCl, 2 mM DTE, 4 µM resazurin, pH = 7) at 70 °C and the final cell protein concentration and sodium formate concentration as indicated

### H_2_ production in batch-operated stirred-tank bioreactors

To avoid pH effects on productivity, formate oxidation was investigated under controlled reaction conditions using batch-operated stirred-tank bioreactors with continuous gas sparging of 100% N_2_. *T. kivui* cells were grown in 20 L of complex medium with glucose as growth substrate to the end of the exponential growth phase, and resting cells were prepared. The bioreactor contained 50 mM imidazole buffer and sodium formate was added to a final concentration of 600 mM. The reaction was started by adding resting cells to a final cell protein concentration of 0.6 mg mL^−1^. Two different approaches were tested in the bioreactor experiments, either keeping the pH value constant at 7.2 by titrating with H_3_PO_4_ (Batch [1]) or by keeping the pH value unaffected during the whole experiment (Batch [2]).

**Fig. 4:**
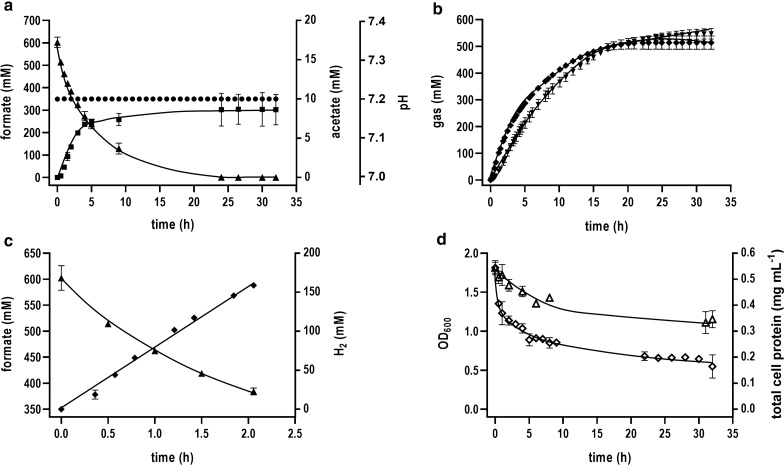
Formate-driven H_2_ production in a batch-operated stirred-tank bioreactor with pH control [Batch 1]. The bioreactor contained imidazole buffer (50 mM imidazole, 20 mM KCl, 2 mM DTE, pH 7.0) at a temperature of 60 °C. The stirrer speed was set at 400 rpm and a continuous gas flow rate of 50 mL min^−1^ with 100% N_2_ was applied. 600 mM of sodium formate was added prior to the start of the experiment. The reaction was started by transferring resting cells to a final total cell protein concentration of 0.6 mg mL^−1^ into the bioreactor and the pH value was kept constant at 7.2 over the whole process by titration with 4 M H_3_PO_4_. **a** The formate consumption, acetate formation and pH curve are shown over time. **b** Gas evolution of H_2_ and CO_2_ during the experiment. **c** Initial formate oxidation and H_2_ production kinetics of the first 2 h. **d** Optical density and total cell protein concentration in the bioreactor during the whole process time. Black triangles up, formate; black squares, acetate; black circles, pH value; black diamonds, H_2_; black triangles down, CO_2_; empty triangles up, total cell protein concentration; empty diamonds, optical density at 600 nm. All data points are mean ± SD, *N* = 2

As seen in Batch [1], formate oxidation started immediately after the addition of the cell suspension to the bioreactor (Fig. 4a) and H_2_ and CO_2_ evolution were also directly observed (Fig. 4b). At the beginning of the experiment, the amount of detected CO_2_ was slightly lower than the amount of H_2_ observed, but similar amounts were observed during the course of the experiment. This observation is caused due to the higher solubility of CO_2_ in the liquid phase compared to H_2_, since both gases are produced in stoichiometric amounts in the catalyzed reaction. Based on the first 2 h and a total cell protein concentration of 0.54 mg mL^−1^, the specific H_2_ productivity and formate oxidation rate were determined to be 147 and 192 mmol g^−1^ h^−1^, respectively (Fig. 4c). After 24 h of process time, 600 mM formate was consumed and 514 mM of H_2_ was produced. Acetate was only formed in small amounts (8.6 ± 1.4 mM), resulting in a high *Y*_(H2/formate)_ of 0.86 mol mol^−1^ as also observed in serum bottle experiments. The pH value was kept constant at 7.2 during the whole process (Fig. 4a) and 316 mM of H_3_PO_4_ was fed into the bioreactor in total. This is in accordance with the expectations since H_3_PO_4_ is able to release two H^+^ at pH 7.2. Unfortunately, optical density as well as total cell protein concentration showed an exponential decay, indicating the lysis of cells over the time (Fig. 4d).

**Fig. 5:**
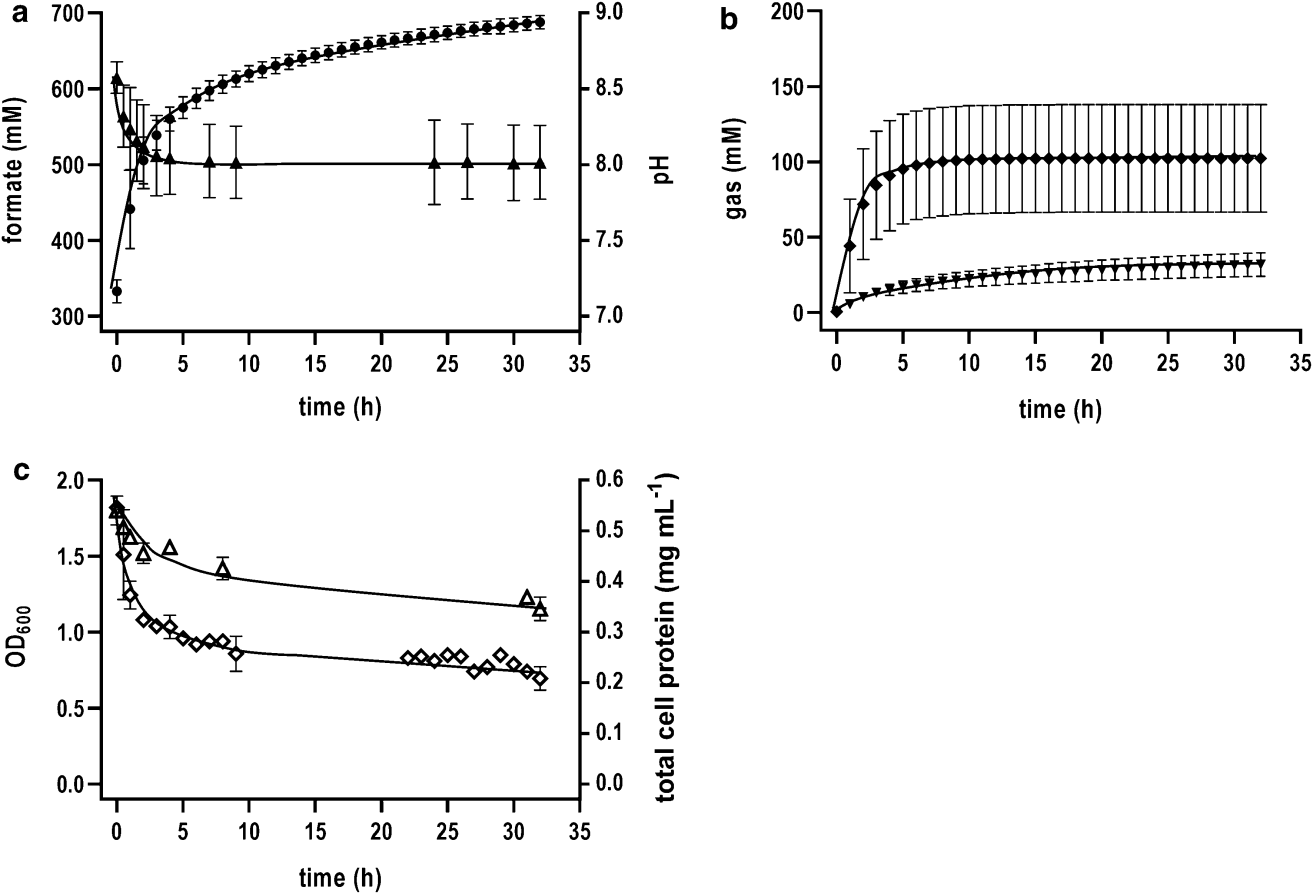
Formate-driven H_2_ production in a batch-operated stirred-tank bioreactor without pH control [Batch 2]. The bioreactor contained imidazole buffer (50 mM imidazole, 20 mM KCl, 2 mM DTE, pH 7.0) at a temperature of 60 °C. The stirrer speed was set at 400 rpm and a continuous gas flow rate of 50 mL min^−1^ with 100% N_2_ was applied. 600 mM of sodium formate was added prior to the start of the experiment. The reaction was started by adding resting cells to a final total cell protein concentration of 0.6 mg mL^−1^ into the bioreactor. The pH value was not controlled. **a** The formate consumption and pH are shown over time. **b** Gas evolution of H_2_ and CO_2_ during the experiment. **c** Optical density and total cell protein concentration in the bioreactor during the whole process time. Black triangles up, formate; black circles, pH value; black diamonds, H_2_; black triangles down, CO_2_; empty triangles up, total cell protein concentration; empty diamonds, optical density at 600 nm. All data points are mean ± SD, *N* = 2

Different results were obtained in the control experiment without pH control (Batch [2]). Formate oxidation and hydrogen production started immediately after cell addition, however, the reaction sharply stopped after 6 h (Fig. 5a, b). At that time point no hydrogen was produced anymore and the formate concentration stayed constant in the bioreactor broth. Looking at the pH curve, the loss in activity seemed to be directly connected to the pH value (Fig. 5a). Due to formate oxidation, the pH value strongly increased from 7.2 to 8.4 in the first 6 h. After the catalytic activity of the cells was diminished, the change in pH slowed down, reaching a pH value of 8.9 after 34 h. This clearly indicates the dependence of the whole cell-based formate-driven hydrogen production on the pH. In contrast to Batch [1], similar amounts of CO_2_ and H_2_ were not observed in the off-gas analysis during the course of the experiment (Fig. 5b). This was based on the fact, that CO_2_ is stored in the liquid phase in form of bicarbonate (HCO_3_^−^) under more alkaline conditions. At pH 8–9 the bicarbonate anion is the predominant form. Optical density as well as total cell protein concentration showed a similar exponential decay as observed in Batch [1] (Fig. 5c).

## Discussion

**Fig. 6:**
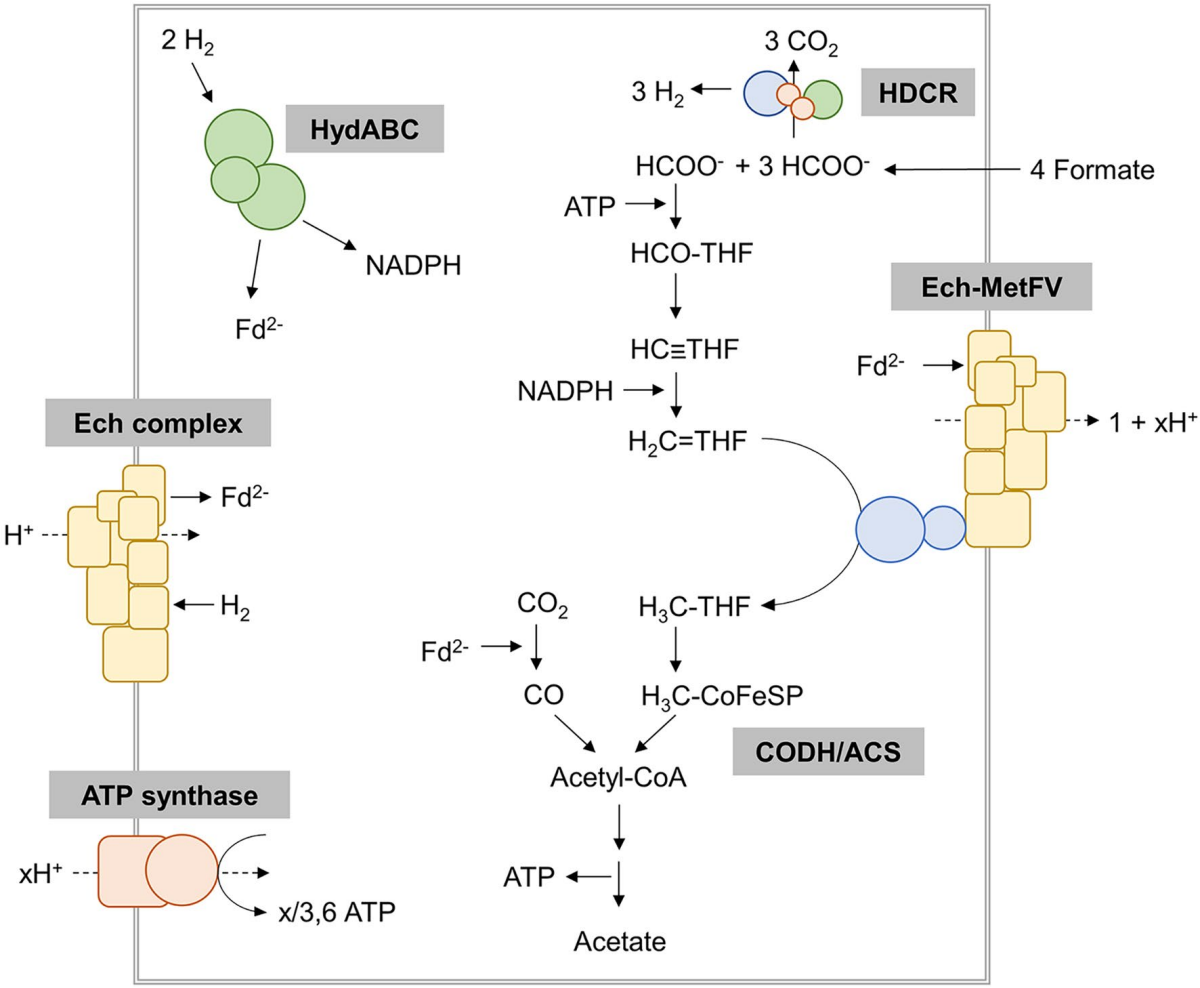
Model of the bioenergetics and biochemistry of acetogenesis from formate in *T. kivui*. HDCR, hydrogen-dependent CO_2_ reductase; HydABC, electron-bifurcating hydrogenase; CODH/ACS, CO dehydrogenase/acetyl-CoA synthase; THF, tetrahydrofolate; HCO-THF, formyl-THF; HC-THF, methenyl-THF; H_2_C-THF, methylene-THF; H_3_C-THF, methyl-THF; Ech, energy-converting hydrogenase; Ech-MetFV, energy-converting hydrogenase complex coupled to methylene-THF reductase; CoFeSP, corrinoid-iron-sulfur-protein; Fd^2−^, reduced ferredoxin. The Ech-MetFV complex is hypothetical. The ion stoichiometries for the membrane proteins have not been determined experimentally. Adapted from Katsyv et al. [55]

In this study, we investigated the biological hydrogen production from formate using the thermophilic acetogenic bacterium *T. kivui* as biocatalyst. Formate-based H_2_ production was catalyzed by the HDCR enzyme. Since acetogenic bacteria can grow under lithoautotrophic conditions, a sufficient CO_2_ reduction system is necessary to fix one CO_2_ molecule in the methyl-branch of the WLP via formate dehydrogenases (CO_2_ reductases). Here, CO_2_ is reduced with two electrons to the intermediate formate which is further converted to acetate (Fig. 6). Different types of purified and characterized formate dehydrogenases of acetogenic bacteria clearly demonstrate the diversity in subunit composition and the diversity of reductants used. Reductants can be reduced ferredoxin as in case of *Clostridium pasteurianum* [34], NADPH as in case of *M. thermoacetica,* [35], a combination of reductants as in case of *Clostridium autoethanogenum* and *Eubacterium callanderi* KIST612 [36, 37], or even molecular H_2_ as shown for the HDCR enzyme complex of *A. woodii* and *T. kivui* [28, 29]. The enzyme of the latter two organisms is not connected to the metabolism by an external cofactor as electron carrier. Additionally, a good reversibility of the HDCR enzyme complex is also required since the HDCR-containing bacteria *A. woodii* and *T. kivui* can also grow with methanol or formate, respectively, as sole carbon and energy source [15, 38, 39]. During organoheterotrophic conditions, the physiological function of the HDCR enzyme is formate oxidation to provide the necessary reducing equivalents for acetogenesis (Fig. 6). However, in both life-styles the main end product is acetate. For this reason, specific ionophores or uncoupling agents were used in previous studies of *A. woodii* and *T. kivui* to decouple the HDCR reaction of CO_2_ reduction from the WLP. By lowering the intracellular amount of ATP, the ATP-dependent further conversion of formic acid to acetic acid in the WLP of acetogenic bacteria is blocked and formate (in case of CO_2_ reduction) or H_2_ and CO_2_ (in case of formate oxidation by *A. woodii*) became the predominant product(s). Interestingly, the situation is different in resting cells of *T. kivui*. Here, no specific ionophore nor metabolic uncoupler were needed to efficiently catalyze the oxidation of formate to H_2_ and CO_2_ by whole cells. Moreover, only small amounts of acetate were produced and a high Y_(H2/formate)_ could be reached. This behavior is highly unusual for formate utilizing acetogens since growth studies demonstrated the formation of acetate as the dominant product (Fig. 6). Putative adaptation and CO_2_ concentration effects could be experimentally excluded. Since the elucidation of the formate metabolism in *A. woodii* and *T. kivui* is still in its infancy and little is known about formate import/export and the corresponding transporters, further metabolic, bioinformatic and genetic studies are necessary to reveal this enigma. However, future metabolic engineering of *T. kivui* by knocking-out responsible genes coding for WLP enzymes downstream of the HDCR reaction could further help to diminish unwanted side product formation in form of acetate. Here, the knock-out of the formyl-THF synthetase genes would be the most obvious way to prevent the further conversion of formic acid towards acetate in the methyl-branch of the WLP.

Nevertheless, the highest volumetric hydrogen production rate measured during the experiments described in this study was 999 mmol L^−1^ h^−1^ (using 4 mg/mL cell protein). This HER is higher than the highest HER described for other wild-type organisms like *A. woodii* (79 mmol L^−1^ h^−1^) [33] or *E. coli* (9.4 g L^−1^ h^−1^, which equals 4660 mmol L^−1^ h^−1^ using 74 mg/mL dry cells) [10]. This study clearly demonstrates the promising potential of *T. kivui* cells in formate-driven H_2_ production. As far as we know, the demonstrated qH_2_ of *T. kivui* cells (685 mmol g^−1^ h^−1^) are also among the highest rates which were so far reported in the literature for wild-type organisms. Looking into the thermophilic world of microbial H_2_-producers, only wild-type cells of the hyperthermophilic archaeon *T. onnurineus* showed more or less comparable specific H_2_ production rates 404 mmol g^−1^ h^−1^ as *T. kivui* cells [40]. Of course, biohydrogen production can even be further stimulated in future by homologous overexpression of the HDCR genes or by the identification and optimization of putative formate transporters to increase formate uptake rates into the cell. Enhanced hydrogen production from formate was already shown in high cell density cultures of *T. onnurineus* and in a *T. onnurineus* strain with a mutated formate transporter caused by adaptive evolution of the organism [41, 42].

Moreover, the HDCR complex can be regarded as a functional production unit to achieve H_2_ formation from formate in non HDCR-containing organism as it was recently shown for *E. coli* [43]. That formate-driven H_2_ production can be achieved with non-formate-dependent H_2_ producer was also demonstrated for genetically engineered *Pyrococcus furiosus* cells, containing the Fdh-Mrp-Mbh enzyme complex of *T. onnurineus* NA1 [44]. Looking at ambient temperatures, *E. coli* is one of the most studied examples for microbial hydrogen production. Resting cells of *E. coli* K-12 (WT) showed a typically low formate-driven H_2_ productivity of 200 mmol g^−1^ h^−1^, but 2.8 times higher production rates could be achieved by overexpressing the FHL genes [10, 45]. Due to this overexpression and the use of high cell concentrations of 93 g/L the HER measured (23.6 g L^−1^ h^−1^, which equals 11,707 mmol L^−1^ h^−1^) was significantly higher than in the wild type [10]. This shows the high potential of genetic engineering for the increase of hydrogen production in *T. kivui*.

Another advantage of using *T. kivui* is that it grows in mineral salt solutions, without addition of yeast extract or other additions such as vitamins. Here, we have used complex medium to grow the cells for the preparation of resting cells, but in further upscaling experiments, mineral medium can be used as well. In addition, cells can be grown on the inexpensive carbon source formate, rather than on glucose, and *T. kivui* even grows on syngas in mineral media [46], which would add, in addition, to a zero-carbon balance.

In this study, stirred-tank bioreactor experiments did not only demonstrate the upscaling feasibility of our applied system, moreover, limitations in closed-batch serum bottle experiments became apparent. Only a fraction of formate could be oxidized in bioreactor experiments without pH control compared to experiments with controlled pH due to the alkalization of the buffer. Since sodium formate (and not the pure acid) was oxidized, the missing protons (H^+^) to form H_2_ were taken up from the liquid phase. Thereby, the proton concentration in the liquid phase decreased and the pH value of the buffer became alkaline. Since the investigated reaction of formate-based H_2_ production is close to the thermodynamic equilibrium, minor adjustments of the reaction conditions can affect the direction of the reaction. For example, if the pH is drifting to a more alkaline value (i.e., pH 8), the Gibbs free energy value gets much more positive (*G* >  > 0; endergonic) and the equilibrium is strongly shifted to the side of the educts (*K* <  < 1). Because of that, the pH value is an important and critical parameter in the investigated reaction which has to be kept in mind to achieve a complete conversion of the substrate formate to the gaseous products H_2_ and CO_2_. Also high growth temperatures and prevailing environmental conditions (i.e., low H_2_ concentrations) change the thermodynamics in favor of formate oxidation and enables for example the hyperthermophilic archaeon *T. onnurineus* to grow at high temperatures by the oxidation of formate to H_2_ + CO_2_ [25]. The thermodynamics of the formate-oxidizing metabolism and the implications for H_2_ production were already discussed in greater detail [40]. In another example, growth of the sulfate-reducing bacterium *Desulfovibrio* *vulgaris* was coupled to formate-driven H_2_ production using a bioreactor with continuous gas sparging and without sulfate availability or syntrophic partners [47]. Enough energy can also be released in the formate oxidation reaction if the H_2_ concentration is kept at a low level. In nature, this can be achieved by the coupling to H_2_-consuming partner organisms which maintain a low-enough H_2_ partial pressure and which was demonstrated in a coculture of *Moorella* sp. strain AMP or *Desulfovibrio* sp. strain with a hydrogen-consuming methanogen [48].

## Conclusion

This study demonstrated that whole cells of *T. kivui* can be used as biocatalysts to catalyze formate oxidation with concomitant evolution of H_2_ and CO_2_. The H_2_ productivity indicates the great potential of *T. kivui* cells for practical application in comparison to other H_2_-producing microbes. Because of the good reversibility of the HDCR reaction, the organism could be a central part in H_2_ storage/CO_2_ capture into formate as well as in hydrogen production from formate. Even if the demonstrated rates are among the highest rates reported, the existing genetic tool box of acetogenic bacteria bears further potential to optimize biohydrogen production by *T kivui* cells, thus, contributing to a future sustainable formate/H_2_ bio-economy.

## Methods

### Organism and cultivation

*Thermoanaerobacter kivui* (DSM 2030) wild type was cultivated at 66 °C under anoxic conditions in 50 mL, 500 mL or 20 L media using 120-mL serum bottles or 1-L/22-L flasks (Glasgerätebau Ochs GmbH, Bovenden-Lenglern, Germany). The medium was prepared under anoxic conditions as described before [49, 50]. Media was either carbonate- and phosphate-buffered complex medium according to Leigh et al. [51] or complex medium without additional NaHCO_3_ which was named as phosphate-buffered complex medium. As growth substrate 28 mM glucose or 200 mM sodium formate was used. Optical densities of growing cultures were determined at 600 nm.

### Growth experiments

Growth experiments were performed in 120-mL serum bottles with 50 mL of the corresponding complex medium at 66 °C under anoxic conditions. Medium used was either carbonate- and phosphate-buffered complex medium with a gas phase of N_2_ + CO_2_ (80:20%[v:v]) or phosphate-buffered complex medium with a N_2_ (100% [v:v]) or N_2_ + CO_2_ (80:20%[v:v]) gas phase. As growth substrate 200 mM of sodium formate was used. These media were inoculated with a glucose-grown culture of *T. kivui* to an optical density of 0.05. Samples were taken every 2 h to determine the optical densities and pH values (pH-meter CG 825, SCHOTT Instruments GmbH, Mainz, Germany). Cells were pelleted by centrifugation (18,000*g*, 5 min, 4 °C) and the supernatant was used to determine acetate and formate concentrations.

### Preparation of resting cells

Resting cells of *T. kivui* were prepared as described before [31]. Glucose-grown cells were harvested at the end of the exponential growth phase at OD 1.7–2.2 and sodium formate-grown cells were harvested at OD 0.4–0.5 by centrifugation. Then, cells were washed two times and resuspended in imidazole buffer (50 mM imidazole, 20 mM MgSO_4_, 20 mM KCl, 2 mM DTE, 4 µM resazurin, pH 7). All preparation steps were done in an anaerobic chamber (Coy Laboratory Products, Grass Lake, MI). For storage at 4 °C a gas-tight Hungate tube with a N_2_ atmosphere (100%) was used. The total cell protein concentration of the cell suspension was determined according to Schmidt et al. [52].

### Cell suspension experiments

To characterize hydrogen production from formate by resting cells of *T. kivui*, 120-mL serum bottles (Glasgerätebau Ochs GmbH, Bovenden-Lenglern, Germany) were used with a total liquid reaction volume of 10 mL under a N_2_ atmosphere (100%). Serum bottles were incubated in a shaking water bath to preheat the corresponding buffer to the temperature as indicated. Prior to the start of the reaction, resting cells were added to the buffer at the concentration as indicated and were incubated for 10 min. When metabolic inhibitors DCCD (*N,N′*-dicyclohexylcarbodiimide), TCS (3,3′,4′,5-tetrachlorosalicylanilide), ETH2120 (*N,N,N´,N´*-tetracyclohexyl-1,2-phenylenedioxydiacetamide; all dissolved in EtOH) were used, they were additionally added to the buffer. In case of O_2_ sensitivity studies, corresponding amounts of atmospheric air (assuming an O_2_ content of 21%) were added to the serum bottle head space and dissolved O_2_ concentrations were calculated according to the law of Henry. Atmospheric pressure was ensured in the serum bottles prior to the start of the experiments. The experiments were started by adding the substrate (sodium, potassium or ammonium formate). If not otherwise stated, generally a temperature of 60 °C, a sodium formate concentration of 150 mM, a protein concentration of 0.6 mg mL^−1^ and imidazole buffer (50 mM imidazole, 20 mM MgSO_4_, 20 mM KCl, 2 mM DTE, 4 µM resazurin, pH 7) was used. A different buffer composition (25 mM MES, 25 mM MOPS, 25 mM HEPES, 25 mM EPPS, 25 mM CHES, 20 mM MgSO_4_, 20 mM KCl, 2 mM DTE, 4 µM resazurin) was used to determine the pH optimum. The pH value of this buffer was set to 5–10 at room temperature. Gas samples were taken with a gas-tight syringe during the experiments to analyze hydrogen concentrations via gas chromatography as described before [28]. Liquid samples were taken over time and were subsequently centrifuged (18,000*g*, 5 min, 4 °C) to remove the cells. The supernatant was frozen till the formate and acetate concentrations were determined.

### Batch experiments in stirred-tank bioreactors

The batch-operated stirred-tank bioreactor experiments were carried out in Biostat Aplus bench-top reactors from Sartorius (Melsungen, Germany) as described before [32]. The working volume was 2 L containing modified imidazole buffer (50 mM imidazole, 20 mM KCl, 2 mM DTE, pH 7). Each bioreactor was equipped with micro-sparger, baffles, two Rushton-impellers, pH-probe (Hamilton, Bonaduz, Switzerland), temperature probe and a redox potential probe (Hamilton, Bonaduz, Switzerland). The temperature of the buffer was maintained at 60 °C, using a cooling finger and heating sleeve. The stirring speed was 400 rpm and a gas flow rate of 50 mL/min with 100% N_2_ was applied using a digital mass-flow controller (Bronkhorst High-Tech, Ruurlo, Netherlands). The bioreactor buffer was prepared under aerobic, non-sterile conditions and oxygen was removed by subsequent sparging with 100% N_2_ for about 16 h. The headspace of the bioreactor was at atmospheric pressure. 600 mM of anoxic sodium formate was added bevor the reaction was started by the addition of resting cells to a final cell protein concentration of 0.6 mg mL^−1^. The pH was either controlled at pH 7.2 via titration with 4 M H_3_PO_4_ during the experiment (Batch [1]) or was not controlled (Batch [2]). Liquid samples (2 mL) were taken at defined time points for HPLC measurement as well as OD and protein determination. The samples were centrifuged (18,000*g*, 8 min, room temperature) to remove cells and the supernatant was frozen at − 20 °C until further off-line analysis.

### Analytical methods

The concentrations of formate and acetate were measured by using a commercially available formic acid and acetic acid determination kit (Boehringer Mannheim/R-Biopharm AG, Mannheim/Darmstadt, Germany) following the instructions of the manufacturer or by high-performance liquid chromatography (HPLC) as described before [53]. Bioreactor off-gas analysis was conducted via a Micro-GC (Inficon, Bad Ragaz, Switzerland) using the analytical conditions and columns as described before [54]. Gas samples from closed-batch serum bottle cell suspension experiments were analyzed via GC as described in [28]. The total cell protein concentration of the prepared cell suspensions was determined according to Schmidt et al. [52].

### Chemicals

All chemicals were supplied by Sigma-Aldrich (St. Louis, USA) and Carl Roth GmbH & Co KG (Karlsruhe, Germany). All premixed gases for cell suspension experiments and bioreactor applications were purchased from Nippon Gases Europe (Düsseldorf, Germany). Pure gases such as N_2_ (purity of 5.0) were purchased from Air Liquide (Paris, France).

## Supplementary Information

**Additional file 1: Figure S1.**Growth of T. kivui on formate in the presence of different concentrations of CO_2_. Complex medium with 200 mM sodium formate was inoculated to an OD of 0.05 with glucose-grown culture of *T. kivui *and cultivated at 66 °C. **a, b) **carbonate/phosphate-buffered medium with N_2_+CO_2_ (80:20%[v:v]) as gas phase, **c, d) **phosphate-buffered medium with N_2_ (100% [v/v]) as gas phase, **e, f) **phosphate-buffered medium with N_2_ + CO_2_ (80:20% [v:v]) as gasphase. OD_600_ (empty circles), pH (black diamonds), formate (black triangles up), acetate (black circles). All data points are mean ± SD, N = 2. **Figure S2. **Effect of different formate concentrations on H_2 _production by formate-grown resting cells of T. kivui. Resting cells (0.6 mg mL^-1^) were added to preheated (60 °C) imidazole buffer (50 mM imidazole, 20 mM MgSO_4_, 20 mM KCl, 2 mM DTE, 4 µM resazurin, pH 7) under a N_2_ atmosphere. The reaction was started by addition of 150 mM (black squares), 300 mM (black triangles up) or 2 M (black triangles down) of sodium formate. The hydrogen concentration is plotted against the time. Acetate was only produced in traces (0.15 ± 0.02 mM). All data points are mean ± SD, N = 2. **Figure S3. **pH dependence of H_2_ production. Resting cells (0.6 mg mL^-1^) were added to preheated (60 °C) buffer (25 mM MES, 25 mM MOPS, 25 mM HEPES, 25 mM EPPS, 25 mM CHES, 20 mM MgSO_4_, 20 mM KCl, 2 mM DTE, 4 µM resazurin) under a N_2_ atmosphere. The pH of the buffer was adjusted to the values of 5 to 10 at room temperature. The reaction was started by addition of 150 mM of sodium formate. Specific H_2_ production rates were calculated based on the first 15 minutes after start of the reaction. All data points are mean ± SD, N = 2. **Figure S4.** Effect of varying temperatures on the H_2_ productivity. Resting cells (0.6 mg mL^-1^) were added to preheated imidazole buffer (50 mM imidazole, 20 mM MgSO_4_, 20 mM KCl, 2 mM DTE, 4 µM resazurin, pH 7) under a N_2_ atmosphere. The reaction was started by adding 150 mM sodium formate. Specific H_2_ production rates were calculated based on the first 15 minutes after start of the reaction. All data points are mean ± SD, N = 2. **Figure S5. **Dependence of the hydrogen production rate on the formate concentration. Resting cells (0.6 mg mL^-1^) were added to preheated (60 °C) imidazole buffer (50 mM imidazole, 20 mM MgSO_4_, 20 mM KCl, 2 mM DTE, 4 µM resazurin, pH 7) under a N_2_ atmosphere. The reaction was started by addition of 25 mM to 8 M of sodium formate. Specific H_2_ production rates were calculated based on the first 10 to 15 minutes after start of the reaction. All data points are mean ± SD, N = 2. **Figure S6.** Influence of the storage time of resting T. kivui cells on the H_2_ productivity. Resting cells (0.6 mg mL^-1^) were added to preheated (60 °C) imidazole buffer (50 mM imidazole, 20 mM MgSO_4_, 20 mM KCl, 2 mM DTE, 4 µM resazurin, pH 7) under a N_2_ atmosphere. The reaction was started by addition of 150 mM of potassium formate. Specific H_2_ production rates were calculated based on the first 15 minutes after start of the reaction. 100% is equivalent to 249 ± 51 mmol g^-1^ h^-1^, N = 23. All data points are mean ± SD, N = 2. **Figure S7.** Effect of different O_**2**_ concentrations on the H_2_ productivity. Resting cells (0.6 mg mL^-1^) were added to preheated (60 °C) imidazole buffer (50 mM imidazole, 20 mM MgSO_4_, 20 mM KCl, 2 mM DTE, 4 µM resazurin, pH 7) under a N_2_ atmosphere. Certain amounts of bottled atmosphere were exchanged with the same volume of air. After incubation for 10 minutes the reaction was started by the addition of 150 mM sodium formate. **a) **Black squares, 0 µM O_2_ (0% [v:v] in head space); black triangles up, 2.75 µM (0.39% O_2_ [v:v]); black triangles down, 5.57 µM O_2_ (0.79% O_2_ [v:v]); black diamonds, 8.3 µM O_2_ (1.2% O_2_ [v:v]); black circles, 11.1 µM O_2_ (1.6% O_2_ [v:v]); empty squares, 13.8 µM O_2_ (1.96% O_2_ [v:v]). **b) **Specific H_2_ production rates under different amounts of O_2_ were calculated based on the first 15 minutes after start of the reaction and plotted against the O_2_ concentration in the liquid phase. All data points are mean ± SD, N = 2.

## Data Availability

All data generated or analyzed during this study are included in this published article and its supplementary information files. If additional information is needed, please contact the corresponding author.

## Abbreviations

H_2_: Hydrogen
CO_2_: Carbon dioxide
O_2_: Oxygen
LOHC: Liquid organic hydrogen carrier
WLP: Wood–Ljungdahl pathway
HDCR: Hydrogen-dependent CO_2_ reductase
THF: Tetrahydrofolate
FHL: Formate hydrogen lyase
ATP: Adenosine triphosphate
Fdh: Formate dehydrogenase
Mbh: Membrane-bound hydrogenase
Mrp: Multisubunit Na^+^/H^+^ antiporter
OD: Optical density at 600 nm
UV/vis: Ultraviolet/visible
K: Equilibrium constant
qH_2_: Specific hydrogen production rate
HER: Volumetric hydrogen production rate
Y_(H2/formate)_: Yield of H_2_ produced per formate consumed
CDW: Cell dry weight
WT: Wild type
t_d_: Doubling time
GC: Gas chromatography
HPLC: High-performance liquid chromatography
rpm: Rounds per minute
DCCD: *N,N′*-Dicyclohexylcarbodiimide
TCS: 3,3′,4′,5-Tetrachlorosalicylanilide
ETH2120: *N,N,N´,N´*-Tetracyclohexyl-1,2-phenylenedioxydiacetamide
DTE: Dithioerythritol
HCO3−: Bicarbonate
MES: 2-Morpholin-4-ylethanesulfonic acid
MOPS: 3-Morpholinopropane-1-sulfonic acid
HEPES: 2-[4-(2-Hydroxyethyl)piperazin-1-yl]ethanesulfonic acid
EPPS: 4-(2-Hydroxyethyl)piperazine-1-propanesulfonic acid
CHES: 2-(Cyclohexylamino)ethanesulfonic acid

## References

[1] Dufour J, Serrano DP, Galvez JL, Moreno J, Garcia C (2009). Life cycle assessment of processes for hydrogen production. Environmental feasibility and reduction of greenhouse gases emissions. Int J Hydrog Energy.

[2] Hay JXW, Wu TY, Juan JC, Md Jahim J (2013). Biohydrogen production through photo fermentation or dark fermentation using waste as a substrate: overview, economics, and future prospects of hydrogen usage. Biofuels Bioprod Biorefin.

[3] Holladay JD, Hu J, King DL, Wang Y (2009). An overview of hydrogen production technologies. Catal Today.

[4] Sinha P, Pandey A (2011). An evaluative report and challenges for fermentative biohydrogen production. Int J Hydrog Energy.

[5] Das D, Veziroǧlu TN (2001). Hydrogen production by biological processes: a survey of literature. Int J Hydrog Energy.

[6] Manish S, Banerjee R (2008). Comparison of biohydrogen production processes. Int J Hydrog Energy.

[7] Rittmann SK, Lee HS, Lim JK, Kim TW, Lee JH, Kang SG (2015). One-carbon substrate-based biohydrogen production: microbes, mechanism, and productivity. Biotechnol Adv.

[8] Rittmann S, Herwig C (2012). A comprehensive and quantitative review of dark fermentative biohydrogen production. Microb Cell Fact.

[9] Ergal I, Fuchs W, Hasibar B, Thallinger B, Bochmann G, Rittmann SKMR (2018). The physiology and biotechnology of dark fermentative biohydrogen production. Biotechnol Adv.

[10] Yoshida A, Nishimura T, Kawaguchi H, Inui M, Yukawa H (2005). Enhanced hydrogen production from formic acid by formate hydrogen lyase-overexpressing *Escherichia coli* strains. Appl Environ Microbiol.

[11] Wang WH, Himeda Y, Muckerman JT, Manbeck GF, Fujita E (2015). CO_2_ hydrogenation to formate and methanol as an alternative to photo- and electrochemical CO_2_ reduction. Chem Rev.

[12] Cotton CA, Claassens NJ, Benito-Vaquerizo S, Bar-Even A (2019). Renewable methanol and formate as microbial feedstocks. Curr Opin Biotechnol.

[13] Yishai O, Lindner SN, de la Gonzalez Cruz J, Tenenboim H, Bar-Even A (2016). The formate bio-economy. Curr Opin Chem Biol.

[14] Drake HL, Küsel K, Matthies C. Acetogenic Prokaryotes. In: The Prokaryotes*.* Edited by Dworkin M, Falkow S, Rosenberg E, Schleifer K-H, Stackebrandt E, vol. 2. New York: Springer; 2006: 373.

[15] Moon J, Dönig J, Kramer S, Poehlein A, Daniel R, Müller V (2021). Formate metabolism in the acetogenic bacterium *Acetobacterium woodii*. Environ Microbiol.

[16] Stephenson M, Stickland LH (1931). Hydrogenase: a bacterial enzyme activating molecular hydrogen: the properties of the enzyme. Biochem J.

[17] Stephenson M, Stickland LH (1932). Hydrogenlyases: Bacterial enzymes liberating molecular hydrogen. Biochem J.

[18] Amend JP, Shock EL (2001). Energetics of overall metabolic reactions of thermophilic and hyperthermophilic *Archaea* and *Bacteria*. FEMS Microbiol Rev.

[19] McDowall JS, Murphy BJ, Haumann M, Palmer T, Armstrong FA, Sargent F (2014). Bacterial formate hydrogenlyase complex. Proc Natl Acad Sci USA.

[20] Hu H, Wood TK (2010). An evolved *Escherichia coli* strain for producing hydrogen and ethanol from glycerol. Biochem Biophys Res Commun.

[21] Kim S, Seol E, Oh Y-K, Wang GY, Park S (2009). Hydrogen production and metabolic flux analysis of metabolically engineered *Escherichia coli* strains. Int J Hydrog Energy.

[22] Kim JY, Jo BH, Cha HJ (2010). Production of biohydrogen by recombinant expression of [NiFe]-hydrogenase 1 in *Escherichia coli*. Microb Cell Fact.

[23] Maeda T, Sanchez-Torres V, Wood TK (2008). Metabolic engineering to enhance bacterial hydrogen production. Microb Biotechnol.

[24] Maeda T, Sanchez-Torres V, Wood TK (2008). Protein engineering of hydrogenase 3 to enhance hydrogen production. Appl Microbiol Biotechnol.

[25] Kim YJ, Lee HS, Kim ES, Bae SS, Lim JK, Matsumi R, Lebedinsky AV, Sokolova TG, Kozhevnikova DA, Cha SS (2010). Formate-driven growth coupled with H_2_ production. Nature.

[26] Lim JK, Mayer F, Kang SG, Müller V (2014). Energy conservation by oxidation of formate to carbon dioxide and hydrogen via a sodium ion current in a hyperthermophilic archaeon. Proc Natl Acad Sci USA.

[27] Mayer F, Lim JK, Langer JD, Kang SG, Müller V (2015). Na^+^ transport by the A_1_A_O_-ATP synthase purified from *Thermococcus onnurineus* and reconstituted into liposomes. J Biol Chem.

[28] Schwarz FM, Schuchmann K, Müller V (2018). Hydrogenation of CO_2_ at ambient pressure catalyzed by a highly active thermostable biocatalyst. Biotechnol Biofuels.

[29] Schuchmann K, Müller V (2013). Direct and reversible hydrogenation of CO_2_ to formate by a bacterial carbon dioxide reductase. Science.

[30] Müller V (2019). New horizons in acetogenic conversion of one-carbon substrates and biological hydrogen storage. Trends Biotechnol.

[31] Schwarz FM, Müller V (2020). Whole-cell biocatalysis for hydrogen storage and syngas conversion to formate using a thermophilic acetogen. Biotechnol Biofuels.

[32] Schwarz FM, Oswald F, Müller V (2021). Acetogenic conversion of H_2_ and CO_2_ into formic acid and *vice versa* in a fed-batch-operated stirred-tank bioreactor. ACS Sustain Chem Eng.

[33] Kottenhahn P, Schuchmann K, Müller V (2018). Efficient whole cell biocatalyst for formate-based hydrogen production. Biotechnol Biofuels.

[34] Scherer PA, Thauer RK (1978). Purification and properties of reduced ferredoxin: CO_2_ oxidoreductase from *Clostridium pasteurianum*, a molybdenum iron-sulfur-protein. Eur J Biochem.

[35] Yamamoto I, Saiki T, Liu SM, Ljungdahl LG (1983). Purification and properties of NADP-dependent formate dehydrogenase from *Clostridium thermoaceticum*, a tungsten-selenium-iron protein. J Biol Chem.

[36] Wang S, Huang H, Kahnt J, Müller AP, Köpke M, Thauer RK (2013). NADP-specific electron-bifurcating [FeFe]-hydrogenase in a functional complex with formate dehydrogenase in *Clostridium autoethanogenum* grown on CO. J Bacteriol.

[37] Dietrich HM, Kremp F, Öppinger C, Ribaric L, Müller V (2021). Biochemistry of methanol-dependent acetogenesis in *Eubacterium callanderi* KIST612. Environ Microbiol.

[38] Kremp F, Poehlein A, Daniel R, Müller V (2018). Methanol metabolism in the acetogenic bacterium *Acetobacterium woodii*. Environ Microbiol.

[39] Jain S, Dietrich HM, Müller V, Basen M (2020). Formate is required for growth of the thermophilic acetogenic bacterium *Thermoanaerobacter kivui* lacking hydrogen-dependent carbon dioxide reductase (HDCR). Front Microbiol.

[40] Lim JK, Bae SS, Kim TW, Lee JH, Lee HS, Kang SG (2012). Thermodynamics of formate-oxidizing metabolism and implications for H_2_ production. Appl Environ Microbiol.

[41] Bae SS, Lee HS, Jeon JH, Lee JH, Kang SG, Kim TW (2015). Enhancing bio-hydrogen production from sodium formate by hyperthermophilic archaeon, *Thermococcus onnurineus* NA1. Bioprocess Biosyst Eng.

[42] Jung HC, Lee SH, Lee SM, An YJ, Lee JH, Lee HS, Kang SG (2017). Adaptive evolution of a hyperthermophilic archaeon pinpoints a formate transporter as a critical factor for the growth enhancement on formate. Sci Rep.

[43] Leo F, Schwarz FM, Schuchmann K, Müller V (2021). Capture of carbon dioxide and hydrogen by engineered *Escherichia coli*: hydrogen-dependent CO_2_ reduction to formate. Appl Microbiol Biotechnol.

[44] Lipscomb GL, Schut GJ, Thorgersen MP, Nixon WJ, Kelly RM, Adams MW (2014). Engineering hydrogen gas production from formate in a hyperthermophile by heterologous production of an 18-subunit membrane-bound complex. J Biol Chem.

[45] Yoshida A, Nishimura T, Kawaguchi H, Inui M, Yukawa H (2007). Efficient induction of formate hydrogen lyase of aerobically grown *Escherichia coli* in a three-step biohydrogen production process. Appl Microbiol Biotechnol.

[46] Weghoff MC, Müller V (2016). CO metabolism in the thermophilic acetogen *Thermoanaerobacter kivui*. Appl Environ Microbiol.

[47] Martins M, Mourato C, Pereira IA (2015). *Desulfovibrio vulgaris* growth coupled to formate-driven H_2_ production. Environ Sci Technol.

[48] Dolfing J, Jiang B, Henstra AM, Stams AJ, Plugge CM (2008). Syntrophic growth on formate: a new microbial niche in anoxic environments. Appl Environ Microbiol.

[49] Hungate RE. A roll tube method for cultivation of strict anaerobes. In: Methods in Microbiology*.* Edited by Norris JR, Ribbons DW, vol. 3b. New York and London: Academic Press; 1969: 117–132.

[50] Bryant MP (1972). Commentary on the Hungate technique for culture of anaerobic bacteria. Am J Clin Nutr.

[51] Leigh JA, Mayer F, Wolfe RS (1981). *Acetogenium kivui*, a new thermophilic hydrogen-oxidizing, acetogenic bacterium. Arch Microbiol.

[52] Schmidt K, Liaaen-Jensen S, Schlegel HG (1963). Die Carotinoide der *Thiorhodaceae*. Arch Mikrobiol.

[53] Schwarz FM, Ciurus S, Jain S, Baum C, Wiechmann A, Basen M, Müller V (2020). Revealing formate production from carbon monoxide in wild type and mutants of Rnf- and Ech-containing acetogens, *Acetobacterium woodii* and *Thermoanaerobacter kivui*. Microb Biotechnol.

[54] Wiechmann A, Ciurus S, Oswald F, Seiler VN, Müller V (2020). It does not always take two to tango: "Syntrophy" *via* hydrogen cycling in one bacterial cell. ISME J.

[55] Katsyv A, Jain S, Basen M, Müller V (2021). Electron carriers involved in autotrophic and heterotrophic acetogenesis in the thermophilic bacterium *Thermoanaerobacter kivui*. Extremophiles.

