# Supplementary material for: Formate-driven H2 production by whole cells of Thermoanaerobacter kivui
Source: Biotechnol Biofuels Bioprod. 2022 May 11;15:48. doi: 10.1186/s13068-022-02147-5 (PMC9097184; doi:10.1186/s13068-022-02147-5)
Supplement: Supplementary file 1 — Additional file 1: Figure S1.Growth of T. kivui on formate in the presence of different concentrations of CO2. Complex medium with 200 mM sodium formate was inoculated to an OD of 0.05 with glucose-grown culture of T. kivui and cultivated at 66 °C. a, b) carbonate/phosphate-buffered medium with N2+CO2 (80:20%[v:v]) as gas phase, c, d) phosphate-buffered medium with N2 (100% [v/v]) as gas phase, e, f) phosphate-buffered medium with N2 + CO2 (80:20% [v:v]) as gasphase. OD600 (empty circles), pH (black diamonds), formate (black triangles up), acetate (black circles). All data points are mean ± SD, N = 2. Figure S2. Effect of different formate concentrations on H2 production by formate-grown resting cells of T. kivui. Resting cells (0.6 mg mL-1) were added to preheated (60 °C) imidazole buffer (50 mM imidazole, 20 mM MgSO4, 20 mM KCl, 2 mM DTE, 4 µM resazurin, pH 7) under a N2 atmosphere. The reaction was started by addition of 150 mM (black squares), 300 mM (black triangles up) or 2 M (black triangles down) of sodium formate. The hydrogen concentration is plotted against the time. Acetate was only produced in traces (0.15 ± 0.02 mM). All data points are mean ± SD, N = 2. Figure S3. pH dependence of H2 production. Resting cells (0.6 mg mL-1) were added to preheated (60 °C) buffer (25 mM MES, 25 mM MOPS, 25 mM HEPES, 25 mM EPPS, 25 mM CHES, 20 mM MgSO4, 20 mM KCl, 2 mM DTE, 4 µM resazurin) under a N2 atmosphere. The pH of the buffer was adjusted to the values of 5 to 10 at room temperature. The reaction was started by addition of 150 mM of sodium formate. Specific H2 production rates were calculated based on the first 15 minutes after start of the reaction. All data points are mean ± SD, N = 2. Figure S4. Effect of varying temperatures on the H2 productivity. Resting cells (0.6 mg mL-1) were added to preheated imidazole buffer (50 mM imidazole, 20 mM MgSO4, 20 mM KCl, 2 mM DTE, 4 µM resazurin, pH 7) under a N2 atmosphere. The reaction was started by adding 150 mM sodium [file 13068_2022_2147_MOESM1_ESM.docx]

**Supplementary Information**

**Formate-driven H_2_ production by whole cells of**

***Thermoanaerobacter kivui***

Yvonne Burger^1^, Fabian M. Schwarz^1^ and Volker Müller^1^*

^1^Department of Molecular Microbiology and Bioenergetics, Institute of Molecular Biosciences, Johann Wolfgang Goethe University, 60438 Frankfurt am Main, Germany

^*^Corresponding author:

Mailing address: Max-von-Laue-Str. 9, 60438 Frankfurt, Germany. Phone 49-6979829507. Fax 49-69-79829306. E-mail: vmueller@bio.uni-frankfurt.de

**Additional file 1**


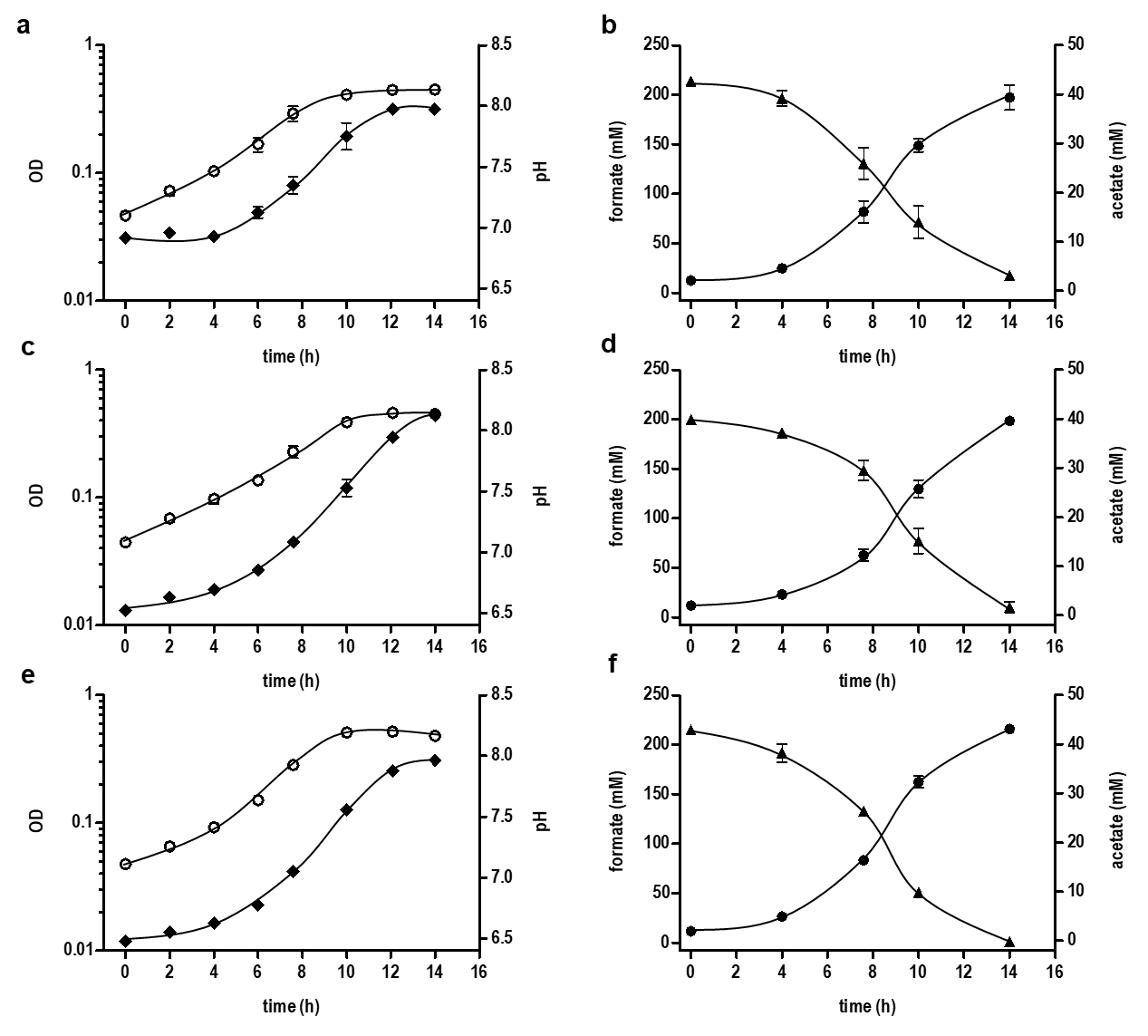


**Figure S1. Growth of *T. kivui* on formate in the presence of different concentrations of CO_2_.** Complex medium with 200 mM sodium formate was inoculated to an OD of 0.05 with glucose-grown culture of *T. kivui* and cultivated at 66 °C. **a, b)** carbonate/phosphate-buffered medium with N_2_+CO_2_ (80:20%[v:v]) as gas phase, **c, d)** phosphate-buffered medium with N_2_ (100% [v/v]) as gas phase, **e, f)** phosphate-buffered medium with N_2_ + CO_2_ (80:20% [v:v]) as gasphase. OD_600_ (empty circles), pH (black diamonds), formate (black triangles up), acetate (black circles). All data points are mean ± SD, N = 2.

**Figure S2. Effect of different formate concentrations on H_2_production by formate-grown resting cells of *T. kivui*.** Resting cells (0.6 mg mL^-1^) were added to preheated (60 °C) imidazole buffer (50 mM imidazole, 20 mM MgSO_4_, 20 mM KCl, 2 mM DTE, 4 µM resazurin, pH 7) under a N_2_ atmosphere. The reaction was started by addition of 150 mM (black squares), 300 mM (black triangles up) or 2 M (black triangles down) of sodium formate. The hydrogen concentration is plotted against the time. Acetate was only produced in traces (0.15 ± 0.02 mM). All data points are mean ± SD, N = 2.


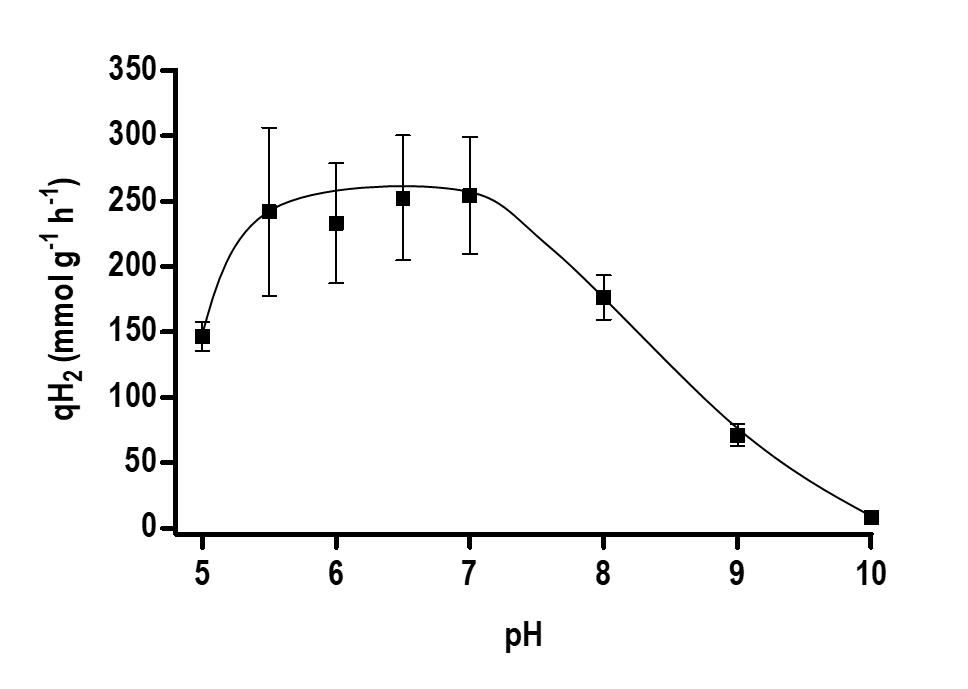


**Figure S3. pH dependence of H_2_ production.** Resting cells (0.6 mg mL^-1^) were added to preheated (60 °C) buffer (25 mM MES, 25 mM MOPS, 25 mM HEPES, 25 mM EPPS, 25 mM CHES, 20 mM MgSO_4_, 20 mM KCl, 2 mM DTE, 4 µM resazurin) under a N_2_ atmosphere. The pH of the buffer was adjusted to the values of 5 to 10 at room temperature. The reaction was started by addition of 150 mM of sodium formate. Specific H_2_ production rates were calculated based on the first 15 minutes after start of the reaction. All data points are mean ± SD, N = 2.


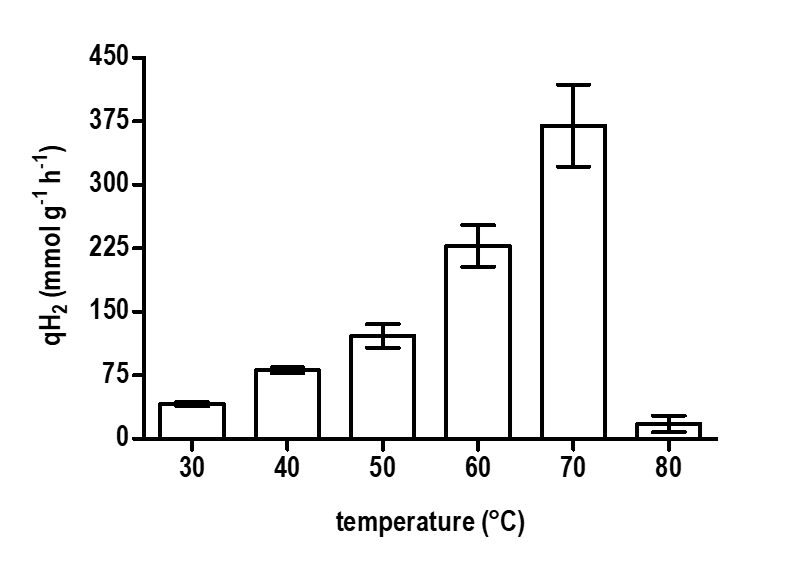


**Figure S4. Effect of varying temperatures on the H_2_ productivity.** Resting cells (0.6 mg mL^-1^) were added to preheated imidazole buffer (50 mM imidazole, 20 mM MgSO_4_, 20 mM KCl, 2 mM DTE, 4 µM resazurin, pH 7) under a N_2_ atmosphere. The reaction was started by adding 150 mM sodium formate. Specific H_2_ production rates were calculated based on the first 15 minutes after start of the reaction. All data points are mean ± SD, N = 2.


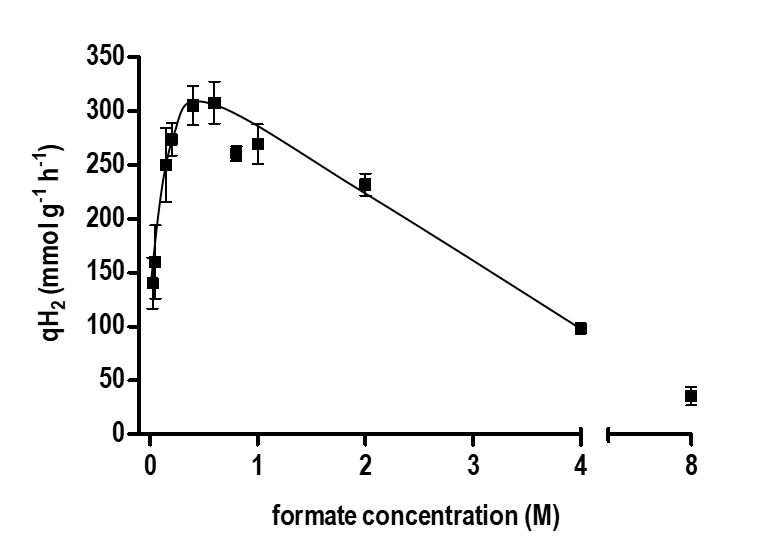


**Figure S5. Dependence of the hydrogen production rate on the formate concentration.** Resting cells (0.6 mg mL^-1^) were added to preheated (60 °C) imidazole buffer (50 mM imidazole, 20 mM MgSO_4_, 20 mM KCl, 2 mM DTE, 4 µM resazurin, pH 7) under a N_2_ atmosphere. The reaction was started by addition of 25 mM to 8 M of sodium formate. Specific H_2_ production rates were calculated based on the first 10 to 15 minutes after start of the reaction. All data points are mean ± SD, N = 2.


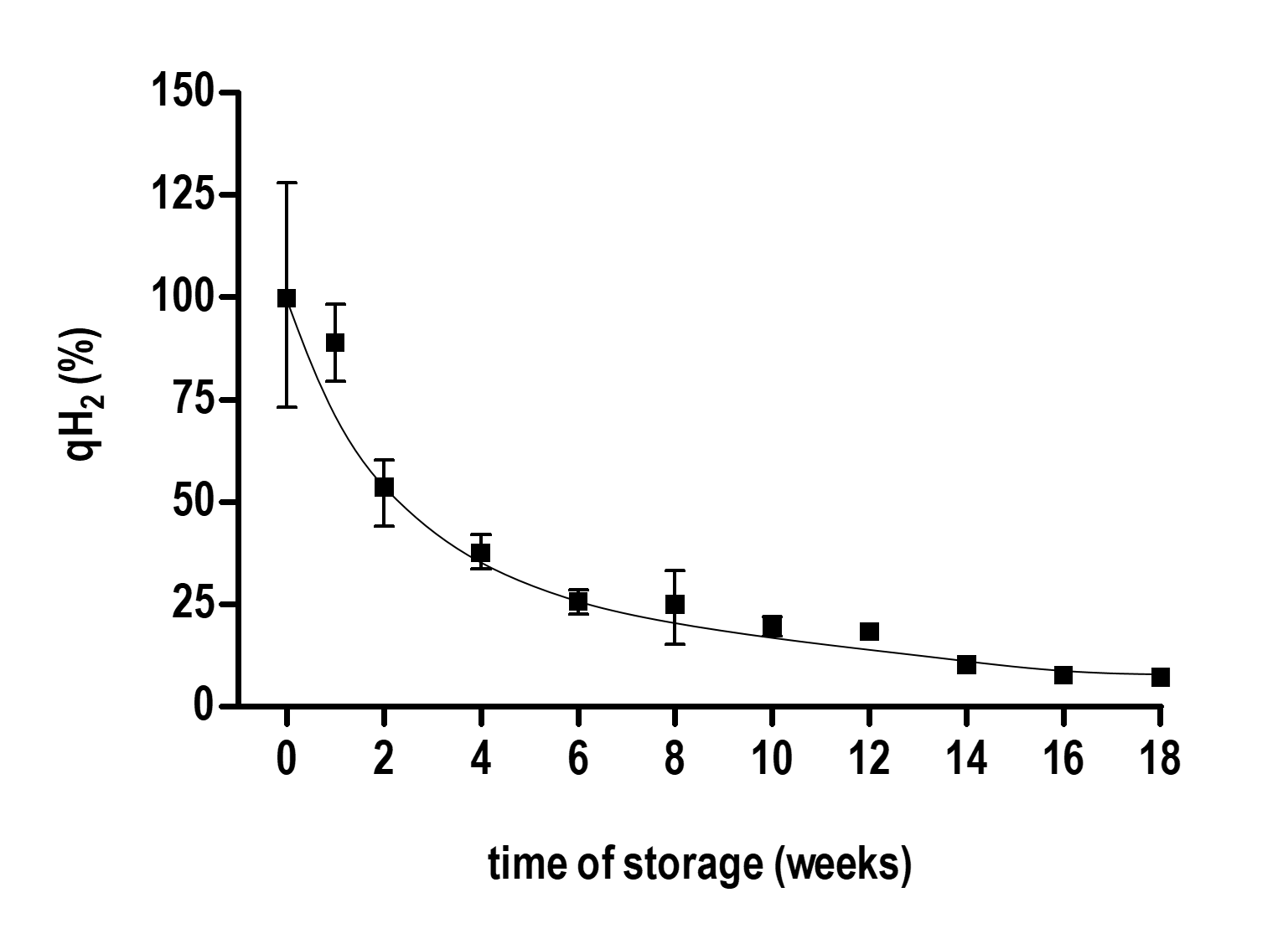


**Figure S6. Influence of the storage time of resting *T. kivui* cells on the H_2_ productivity.** Resting cells (0.6 mg mL^-1^) were added to preheated (60 °C) imidazole buffer (50 mM imidazole, 20 mM MgSO_4_, 20 mM KCl, 2 mM DTE, 4 µM resazurin, pH 7) under a N_2_ atmosphere. The reaction was started by addition of 150 mM of potassium formate. Specific H_2_ production rates were calculated based on the first 15 minutes after start of the reaction. 100% is equivalent to 249 ± 51 mmol g^-1^ h^-1^, N = 23. All data points are mean ± SD, N = 2.


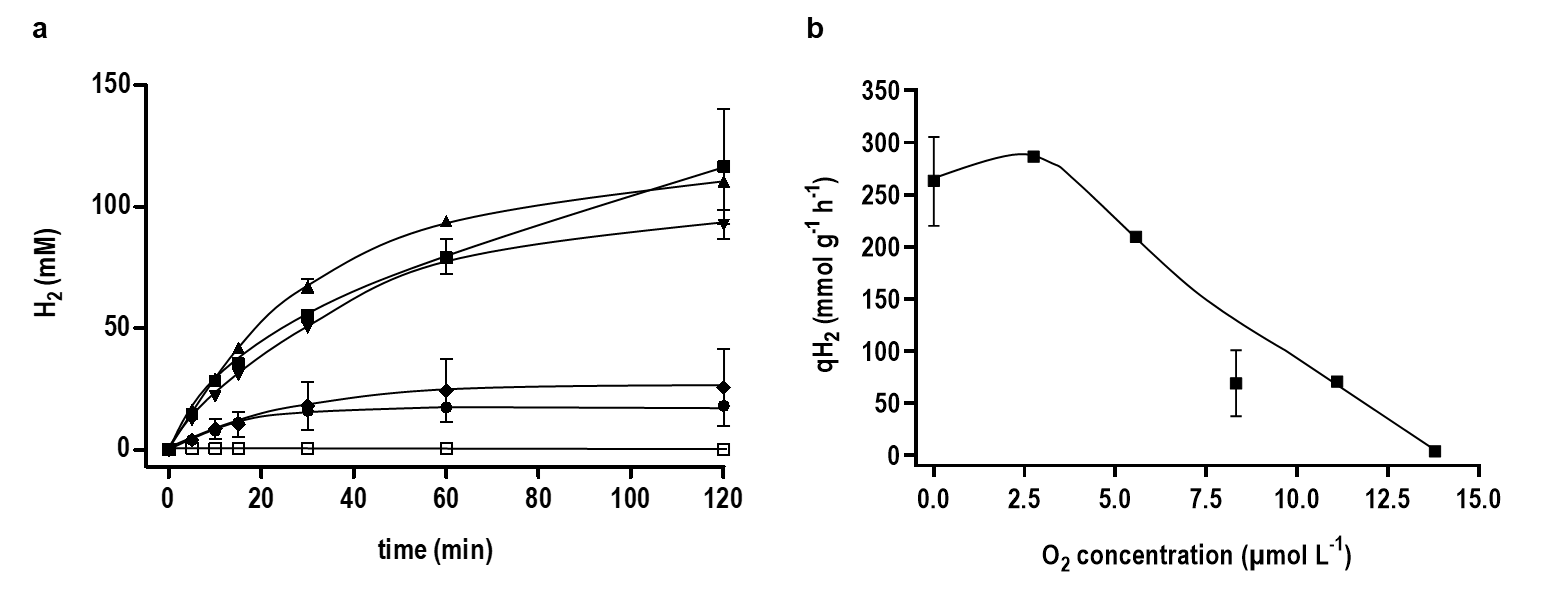


**Figure S7.** **Effect of different O_2_ concentrations on the H_2_ productivity.** Resting cells (0.6 mg mL^-1^) were added to preheated (60 °C) imidazole buffer (50 mM imidazole, 20 mM MgSO_4_, 20 mM KCl, 2 mM DTE, 4 µM resazurin, pH 7) under a N_2_ atmosphere. Certain amounts of bottled atmosphere were exchanged with the same volume of air. After incubation for 10 minutes the reaction was started by the addition of 150 mM sodium formate. **a)** Black squares, 0 µM O_2_ (0% [v:v] in head space); black triangles up, 2.75 µM (0.39% O_2_ [v:v]); black triangles down, 5.57 µM O_2_ (0.79% O_2_ [v:v]); black diamonds, 8.3 µM O_2_ (1.2% O_2_ [v:v]); black circles, 11.1 µM O_2_ (1.6% O_2_ [v:v]); empty squares, 13.8 µM O_2_ (1.96% O_2_ [v:v]). **b)** Specific H_2_ production rates under different amounts of O_2_ were calculated based on the first 15 minutes after start of the reaction and plotted against the O_2_ concentration in the liquid phase. All data points are mean ± SD, N = 2.
